# Prophylactic nicotinamide treatment protects from rotenone-induced neurodegeneration by increasing mitochondrial content and volume

**DOI:** 10.1186/s40478-024-01724-z

**Published:** 2024-03-01

**Authors:** Amin Otmani, Gauti Jóhannesson, Rune Brautaset, James R. Tribble, Pete A. Williams

**Affiliations:** 1 Department of Clinical Neuroscience, Division of Eye and Vision, St. Erik Eye Hospital, Karolinska Institutet, 171 64 Stockholm, Sweden; 2 Department of Clinical Sciences, Ophthalmology, Umeå University, Umeå, Sweden; 3 Wallenberg Centre of Molecular Medicine, Umeå University, Umeå, Sweden; 4 Department of Ophthalmology, University of Iceland, Reykjavik, Iceland

**Keywords:** Nicotinamide, Retina, Retina ganglion cells, Mitochondria, Neurodegeneration, Optic nerve

## Abstract

Leber’s hereditary optic neuropathy (LHON) is driven by mtDNA mutations affecting Complex I presenting as progressive retinal ganglion cell dysfunction usually in the absence of extra-ophthalmic symptoms. There are no long-term neuroprotective agents for LHON. Oral nicotinamide provides a robust neuroprotective effect against mitochondrial and metabolic dysfunction in other retinal injuries. We explored the potential for nicotinamide to protect mitochondria in LHON by modelling the disease in mice through intravitreal injection of the Complex I inhibitor rotenone. Using MitoV mice expressing a mitochondrial-tagged YFP in retinal ganglion cells we assessed mitochondrial morphology through super-resolution imaging and digital reconstruction. Rotenone induced Complex I inhibition resulted in retinal ganglion cell wide mitochondrial loss and fragmentation. This was prevented by oral nicotinamide treatment. Mitochondrial ultrastructure was quantified by transition electron microscopy, demonstrating a loss of cristae density following rotenone injection, which was also prevented by nicotinamide treatment. These results demonstrate that nicotinamide protects mitochondria during Complex I dysfunction. Nicotinamide has the potential to be a useful treatment strategy for LHON to limit retinal ganglion cell degeneration.

**Supplementary Information:**

The online version contains supplementary material available at 10.1186/s40478-024-01724-z.

## Introduction

Neurodegenerative disease accounts for a large and increasing health and economic burden worldwide [1]. In the EU, neurodegenerative disease accounts for ~ 20% of total deaths and in 2017 accounted for ~ 21 million disability-adjusted life-years [2]. Ocular neurodegenerative diseases make up a large proportion of neurodegenerative diseases (*e.g.,* glaucoma is the leading cause of irreversible blindness effecting > 80 million people [3]). Retinal ganglion cells are the output neuron of the retina, the axons of which make up the optic nerve. Under normal physiological conditions retinal ganglion cells do not have the intrinsic capacity to regenerate, and as such, their loss may eventually result in irreversible blindness. Retinal ganglion cells are highly bioenergetic and are intrinsically susceptible to mitochondrial dysfunction and metabolic failure [4]. A decrease in metabolic activity often comes with a concomitant increase in mitochondrial stress [5, 6], which, when combined with other cofactors (*e.g.,* genetic, environmental, other systemic diseases), leads to neurodegeneration. Interestingly, disease-causing mutations in mitochondrial protein coding genes are prevalent in the human population and are present throughout the majority of cell-types in the body. Yet, abnormalities in these genes predominantly affect retinal ganglion cells and present in the form of blinding disorders that have little or no overt extra-ophthalmic pathology (*e.g.,* mitochondrial optic neuropathies such as autosomal dominant optic atrophy (ADOA) caused by mutations in *OPA1* and Leber’s hereditary optic neuropathy (LHON) caused by mtDNA mutations affecting mitochondrial Complex I [7]). In addition to these primary blinding diseases, retinal ganglion cells are an affected population in many neurodegenerative diseases, in addition to the primary pathology, such as Alzheimer’s disease, Parkinson’s disease, and demyelinating diseases [8]. This further supports a hypothesis in which retinal ganglion cells are intrinsically reliant on a tight metabolic balance that can be upset due to other local or systemic stresses.

We have previously identified mitochondrial/metabolic dysfunction and morphological abnormalities affecting the retina and optic nerve at timepoints prior to gross neurodegeneration in glaucoma [5, 9, 10], in addition to Alzheimer’s disease [11, 12]. These findings have been demonstrated to correlate to findings in human patients and human post-mortem tissue [13]. Our studies in glaucoma identified that the capacity to maintain NAD pools (nicotinamide adenine dinucleotide; an essential REDOX cofactor and metabolite) declines in the retina in an age- and intraocular pressure-dependent manner and renders retinal ganglion cells susceptible to glaucoma-related stress, driving glaucomatous neurodegeneration [5, 10]. Preventing NAD depletion via administration of nicotinamide (NAM; the amide of vitamin B_3_; an NAD precursor) potently arrests metabolic decline, prevents glaucoma in animal models [5, 10, 14, 15], and improves visual function in existing glaucoma patients [16, 17].

Given this strong therapeutic potential for nicotinamide, we assessed whether oral nicotinamide could overcome a primary mitochondrial disease, LHON. LHON is a maternally inherited optic neuropathy typically affecting young males. LHON is caused by mtDNA mutations affecting Complex I (the most common being *m.11778G.A/MTND4*, *m.3460G.A/MT-ND1*, *m.14484 T.C/MT-ND6* [18]). This loss of function results in a progressive retinal ganglion cell dysfunction, and eventually, irreversible retinal ganglion cell loss and blindness. There are no long-term neuroprotective agents for LHON, and thus this represents an unmet therapeutic need. To model LHON we utilized a Complex I inhibition mouse model via intravitreal injection of rotenone [19], a natural NADH:ubiquinone reductase (Complex I) inhibitor. Oral nicotinamide is a robust neuroprotective agent for retinal ganglion cells protecting against mitochondrial loss and mitochondrial ultrastructural abnormalities [5, 10] in glaucoma. Given the clinical potential of nicotinamide in glaucoma [16, 17], this supports clinical trial testing for nicotinamide in LHON patients.

## Materials and methods

### Mouse strain, breeding, and husbandry

Mice were bred and experiments were performed in accordance with the Association for Research for Vision and Ophthalmology Statement for the Use of Animals in Ophthalmic and Research. Individual study protocols were approved by Stockholm’s Committee for Ethical Animal Research (10389–2018). Animals were housed and fed in a 12 h light/12 h dark cycle with food and water available ad libitum. C57BL/6 J and MitoV [10] mouse strains were bred and utilized at 8–10 weeks of age. For animal group that were treated with NAM, NAM was dissolved in drinking water to achieve a dose of ~ 500 mg/kg/d (based on average water consumption). Water was protected from light and changed every 3–5 days.

### Intravitreal rotenone model

To model a loss of Complex I activity, as in LHON, we used intravitreal injection of rotenone. This model has previously been described [10, 19, 20]. Animals were anesthetized using an intraperitoneal injection of Ketamine (37.5 mg/kg) and Midazolam (Dormitol, 1.25 mg/kg). Bilateral intravitreal injections were performed using a NanoFil 10 μl glass syringe with a 33G needle (WPI). Mice were injected with either 1.5 μL of 10 mM rotenone (MP Biochemicals) in DMSO (Sigma Life science) or 1.5 μL of DMSO only (control). The NAM treated groups underwent one-week of pre-treatment with NAM prior to injection and continuing until mice were euthanized. Mice were euthanized 24 h after intravitreal injection by cervical dislocation and tissues were collected for analysis.

### Intravitreal virus injection

Mice were anesthetized as above and intravitreal injection performed using the same equipment. Mice were bilaterally injected intravitreally with 1.5 μL of AAV2-CMV-mCherry (AAV-mCherry; Vectorbiolabs) at 2.7 × 10^11^ genome copies/mL. Mice were injected with AAV2-mCherry 3 weeks prior to rotenone/DMSO injection.

### Cryo-sectioning

Optic nerves were fixed for 24 h in a 3.7% PFA in PBS before immersion in 30% sucrose solution for 24 h. Optic nerves were then embedded in O.C.T. (Sakura) and frozen using dry ice before sectioning (20 μm thickness) in the longitudinal axis using a cryostat (Cryostar NX70, Thermo Scientific). Sections were collected on Superfrost Plus slides (Thermo Scientific) and stored at − 20 °C.

### Immunofluorescent labelling

Cryosections and flat mounted retina were subject to immunofluorescent labelling. Cryosections were air dried for 5 min and rehydrated in 1 M PBS for 5 min before following the protocol. Wells were drawn with a hydrophobic barrier pen (VWR), and tissue was permeabilized with 0.5% Triton X-100 (VWR) in 1 M PBS for 20 min, blocked in 1% bovine serum albumin (Fisher Scientific) in 1 M PBS for 45 min, and primary antibody applied and maintained overnight at 4 °C. Primary antibodies used were: anti-RBPMS (Novus, NBP2-20,112, Rabbit, used at 2.2 µg/mL; an RGC specific marker in the retina used only for flat-mounts), anti-GFP (Abcam, ab13970, Chick, used at 20 µg/mL; targeting YFP in MitoV mice), anti-mCherry (Abcam, ab232341, Rabbit, used at 2.02 µg/mL; targeting mCherry in AAV injected mice, used only in optic nerve sections). After 8 washes (15 min each) with 1 M PBS the secondary antibodies were added and incubated at room temperature for 3 h. To limit non-specific binding, 500 µl aliquots of secondary antibodies (at 200 µg/mL) were pre-incubated with a whole PFA-fixed mouse retina for > 48 h. From these aliquots, secondary antibody solutions were prepared at 4 µg/mL for immunofluorescent labelling. Secondary Antibodies used were AF488 (Invitrogen, A11039, goat anti-chick) and AF568 (Invitrogen, A11011, goat anti-rabbit). Secondary only slides were also prepared for all tissues analyzed where only PBS was used (*i.e.* no primary antibody). Tissues were washed (7 × 15 min) as before and DAPI nuclear stain (1 μg/ml in 1 M PBS) was applied for 10 min. Following a last wash in PBS, glass coverslips were mounted using Fluoromount-G (Invitrogen). Slides and coverslips were sealed with nail-varnish.

### Quantification of RGC death

RGC death was quantified in flat-mount retina from MitoV mice (*n* = 6 eyes for all conditions: DMSO control, DMSO NAM, rotenone control, and rotenone NAM). Images were acquired on an epifluorescent microscope (Leica DMi8). Images were acquired at 40X magnification, (0.25 μm/pixel) and were taken at ~ 1000 μm eccentricity from the optic nerve head. Image locations were at 0, 2, 4, 6, 8, and 10 o’clock about the optic nerve head. Images were cropped to 0.01 mm^2^ for cell counting. RBPMS + cells and round DAPI nuclei (*i.e.,* not vascular epithelium) were counted using the cell counter plugin for FIJI [21]. For all metrics an average of the 6 crops was calculated for each retina.

### Super-resolution fluorescent imaging of mitochondria and morphological analysis

Super-resolution fluorescent images of mitochondria were captured using Airyscan2 imaging on a Zeiss LSM980-Airy (40X/1.2 W, 1,7 X optical zoom, 123.59 × 123.59 μm images, 49 nm/pixel, z-stacks with 19 nm slice thickness). As previously [10], we used Alexa Fluor 488 conjugated secondary antibodies targeting YFP in order to limit signal bleaching of YFP when acquiring image volumes. In flatmount retinas, only the Alexa Fluor 488 signal was captured by Airyscan imaging and RBPMS and DAPI were captured as single slice snaps for reference. In optic nerves, both Alexa Fluor 488 signal (YFP mitochondria) and Alexa Fluor 568 signal (mCherry axons) were acquired by Airyscan imaging. Secondary antibody only controls were used for all experiments to set suitable imaging parameters. For flatmount retinas (*n* = 6 for all conditions), 2 images were acquired at 1000 μm eccentricity from the optic nerve head. Images were captured as z-stacks from above the NFL to the lower boundary of the IPL (thus capturing all RGC relevant layers). Ganglion cell layer (GCL)/retinal nerve fiber layer (NFL) and IPL were digitally separated as crops for separate analysis. In the retina and optic nerve, mitochondria were reconstructed in 3D using Imaris software (version 9.3.1). Volume reconstructions were performed using the surfaces tool. We used a surface detail of 0.05 µm and a background subtraction of 3 µm for both NFL/GCL and IPL analysis. Signal intensity was subject to a threshold to remove pixels < 800 for the GCL and < 80 for the IPL, followed by an area filter of 0.1. To exclude noise, volumes < 125 voxels were filtered and removed from subsequent analysis. Data for the total number of mitochondria, and the volume, surface area, and sphericity of individual mitochondria were exported. The average value for each of these metrics was calculated per retina. In optic nerves, mCherry filled axons were reconstructed using the surfaces tool and this was used as a mask to exclude mitochondria outside of this axon volume. This allowed the analysis of mitochondria within individual axon bundles.

### Electron microscopy

Whole eyes and optic nerves were fixed in 2.5% glutaraldehyde and 1% formaldehyde in 0.1 M phosphate buffer, pH 7.4 at room temperature for 1 h and then stored at + 4 °C. After fixation the samples were rinsed in 0.1 M phosphate buffer pH 7.4 prior to post-fixation in 2% osmium tetroxide in 0.1 M phosphate buffer, pH 7.4 at 4 °C for 2 h. The samples were then stepwise dehydrated in ethanol, followed by acetone and finally resin embedded in LX-112 (Ladd Research). Ultrathin sections (~ 80 nm) were prepared using an EM UC7 microtome (Leica). Whole eyes were sectioned in the sagittal plane to obtain sections through the central retina and optic nerve head (the central optic nerve vessel was used as an anatomic landmark). Cross-sections of optic nerve were collected from the end proximal to the eye. Sections were contrasted with uranyl acetate followed by Reynolds lead citrate. TEM imaging was performed in a Tecnai 12 Spirit BioTwin transmission electron microscope (FEI Company) operated at 100 kV and digital images were acquired using a 2kx2k Veleta CCD camera (Olympus Soft Imaging Solutions). Images of the retina (5 to 25 µm distance from the ONH) were acquired in a series from the inner limiting membrane (ILM) to the lower border of the IPL at 26,500 × magnification. Images of optic nerve were acquired with the same magnification as the retinal images, along two lines across the optic nerve separated by a 90-degree angle.

### Ultra-structural measurement of mitochondria

EM images were processed using FIJI. Mitochondria in the NFL/GCL, IPL, and optic nerve were analyzed. For each mitochondrion, multiple morphological metrics were measured: the ratio of the long and short axis length, outer perimeter length, total surface area, cristae number, cristae surface area, the ratio of the cristae surface area to the total surface area, and the mitochondria integrity index. The long and short axis length were measured using the line section tool. The outer perimeter length, total surface area and cristae surface area were measured using the freehand selection tool. The mitochondria integrity index is an index manually measured by counting the number of crossings between the long axis and the cristae [22]. Average values per retina/optic nerve were calculated for all metrics. In the NFL/GCL the number of individual mitochondria were analyzed by condition were: *n* = 6 DMSO control, *n* = 6 DMSO NAM, *n* = 3 rotenone control, *n* = 6 rotenone NAM. In the IPL the number of individual mitochondria were analyzed by condition were: *n* = 6 DMSO control, *n* = 6 DMSO NAM, *n* = 3 rotenone control, *n* = 6 rotenone NAM. In the optic nerve the number of individual mitochondria were analyzed by condition were: *n* = 6 DMSO control, *n* = 6 DMSO NAM, *n* = 6 rotenone control, *n* = 6 rotenone NAM.

### Statistical analysis

The statistical analyses were performed in R. Data were tested for normality with a Shapiro Wilk test. Normally distributed data were compared by One-way ANOVA (with Tukey’s HSD). Non-normally distributed were compared by a Kruskal–Wallis test followed Dunn’s tests with Benjamini and Hochberg correction. When comparing individual mitochondrial morphologies (fluorescent or EM) a linear mixed effects model approach [23, 24] was used to account for the higher-level grouping of data (i.e. multiple mitochondria from a single retina) which limits *P* value inflation whilst limiting the dilution of the heterogeneity of mitochondria within a sample. Conditions were compared individually using this model (*lme4* package [25]) and *P* values were obtained for regression coefficients using the *car* package. Unless otherwise stated, * = *P* < 0.05, ** = *P* < 0.01, ***P < 0.001, NS = non-significant (*P* > 0.05). For box plots, the center hinge represents the median with upper and lower hinges representing the first and third quartiles; whiskers represent 1.5 times the interquartile range.

## Results

### Nicotinamide provides retinal ganglion cell neuroprotection

**Fig. 1:**
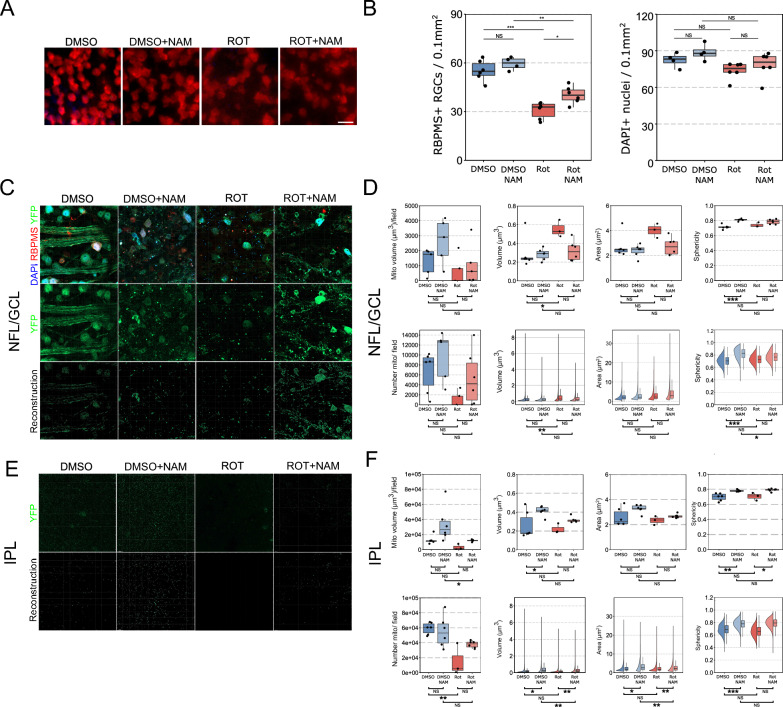
Nicotinamide limits mitochondrial morphological changes in the retina following inhibition of complex I. (**A**) Mice received intravitreal injection of rotenone (ROT) or vehicle control (DMSO). Mice were either pretreated with NAM or remained untreated. (**B**) Significant RGC (RBPMS +) degeneration occurs 1 day after injection of rotenone, in the absence of gross loss of GCL nuclei (DAPI +), supporting a critical vulnerability of RGCs to complex I loss. NAM provides a partial neuroprotection against this loss of RGCS. (**C**) In MitoV mice, high resolution imaging of the NFL/GCL allowed RGC specific YFP + mitochondria in RGC axons and somas to be captured for 3D reconstruction. (**D**) Morphological analysis (average of all mitochondria per retina in box and whisker plots; individual mitochondria/aggregates in raincloud plots) of these mitochondria revealed that rotenone induced a significant increase in mitochondrial volume, likely from the aggregation of damaged mitochondria in the soma. NAM treatment prevented these changes, with morphological features more reflective of normal controls (DMSO). (**E**) High resolution imaging of the IPL allowed RGC specific YFP + mitochondria in RGC dendrites to be captured for 3D reconstruction. (**F**) Morphological analysis demonstrated a reduction in mitochondria numbers in the IPL following rotenone injection, in the absence of detectable changes to morphology. NAM treatment in the absence of complex 1 inhibition (DMSO + NAM) resulted in larger mitochondria in the IPL. Scale bar = 20 µm in A. * *P* < 0.05, ** *P* < 0.01, *** *P* < 0.001, NS = *P* > 0.05

We have previously demonstrated that nicotinamide can overcome age- and intraocular pressure-dependent stress in retinal ganglion cells [5, 10]. Using a well-established model of Complex I inhibition (intravitreal rotenone injection) which results in acute mitochondrial metabolic stress and retinal ganglion cell loss, we first assessed whether oral nicotinamide could prevent gross retinal ganglion cell loss in this model. Mice received NAM 1 week prior to rotenone injection or were untreated. 24 h post-rotenone injection (abbreviated to ROT in figures), 61.4% of retinal ganglion cells survived compared to 71.5% (*P* < *0.04*) in nicotinamide treated mice (500 mg/kg/d, equivalent to ~ 2 g/d in human, two-thirds of the current glaucoma clinical trial doses). We did not detect any significant changes in the density of DAPI nuclei across any conditions (Fig. 1A, B). These data suggest that a prophylactic treatment with NAM at 500 mg/kg/d provides a significant protection against the metabolic stress generated by rotenone in vivo after one week of pre-treatment and confirm the reproducibility of the results in an LHON-relevant disease model.

### Complex I inhibition causes aggregation of mitochondria in the GCL/NFL and fragmentation in the IPL which is protected against by nicotinamide

To assess gross mitochondrial changes specifically in retinal ganglion cells, we utilized our recently published mitochondrial reporter strain, MitoV [10], in which only retinal ganglion cells express fluorescent mitochondria in the inner retina. A major benefit in these mice is the capacity to quantitatively study the global mitochondrial distribution in retinal ganglion cells during pathological processes and to simultaneously acquire a large number (100,000 s) of mitochondria per condition and treat these images in a semi-automated manner using 3D reconstruction software. To investigate the effect of nicotinamide on mitochondrial networks following Complex I inhibition, we optically transected retinal ganglion cell compartments from super resolution confocal images of whole mounted retinas (Fig. 1C). Retinal ganglion cell specific YFP + mitochondria were imaged and reconstructed from 2 distinct retinal ganglion cell compartments: (1) the nerve fiber layer (NFL; RGC axons)/ganglion cell layer (GCL; retinal ganglion cell somas) and (2) the inner plexiform layer (IPL; retinal ganglion cell dendrites). In the NFL/GCL, the total mitochondrial volume was not significantly altered by rotenone and there was no significant change to the total number of mitochondria although this trended towards being lower. The average volume of mitochondria increased following rotenone injection (as an average per retina, and at an individual mitochondrial level), but surface area and sphericity did not change (Fig. 1D). This suggests that under acute mitochondrial stress, surviving mitochondria either become enlarged or aggregate (into volumes that cannot be optically dissected at this resolution). In animals prophylactically treated with nicotinamide prior to rotenone injection, these changes were partially mitigated. Mitochondrial volume, surface area, and sphericity were not significantly different compared to untreated rotenone injected eyes (ROT), or in comparison to vehicle only controls prophylactically treated with nicotinamide (DMSO + NAM) but trended towards an average morphology between these two conditions (Fig. 1D). Nicotinamide treatment in the absence of any insult (DMSO + NAM) resulted only in a significant increase in mitochondrial sphericity (as an average per retina, and at an individual mitochondrial level) compared to untreated controls (DMSO) (Fig. 1D). These results demonstrate that nicotinamide may partially prevent increases to mitochondrial volume caused by Complex I inhibition in retinal ganglion cell soma and axons, if given prophylactically.

Retinal ganglion cell specific YFP + mitochondria in the inner plexiform layer (IPL; retinal ganglion cell dendrites) demonstrated a different response to those in the NFL/GCL. The total volume of mitochondria was not significantly altered by rotenone, but there was a significant decrease in the total number of mitochondria (Fig. 1E). This decrease was prevented by nicotinamide (ROT + NAM) supporting a hypothesis that at least a component of the protective mechanism of nicotinamide is due to an increased mitochondrial population in the IPL. In ROT eyes, remaining mitochondria demonstrated no significant changes to morphology compared to DMSO, but those treated with nicotinamide (ROT + NAM) demonstrated an increase in global mitochondrial sphericity (as an average per retina) and increased individual mitochondrial volume and surface area relative to untreated rotenone eyes (ROT). Nicotinamide treatment in the absence of any insult (DMSO + NAM) resulted in an increase in individual mitochondrial volume and surface area relative to DMSO only controls. In DMSO + NAM, mitochondria were also significantly more voluminous and had a greater surface area than in ROT + NAM (Fig. 1E). These data suggest that in the IPL, NAM results in larger mitochondria, which mitigates Complex I inhibition-induced mitochondrial shrinkage.

### Nicotinamide increases total mitochondria within retinal ganglion cells axons in the optic nerve

**Fig. 2:**
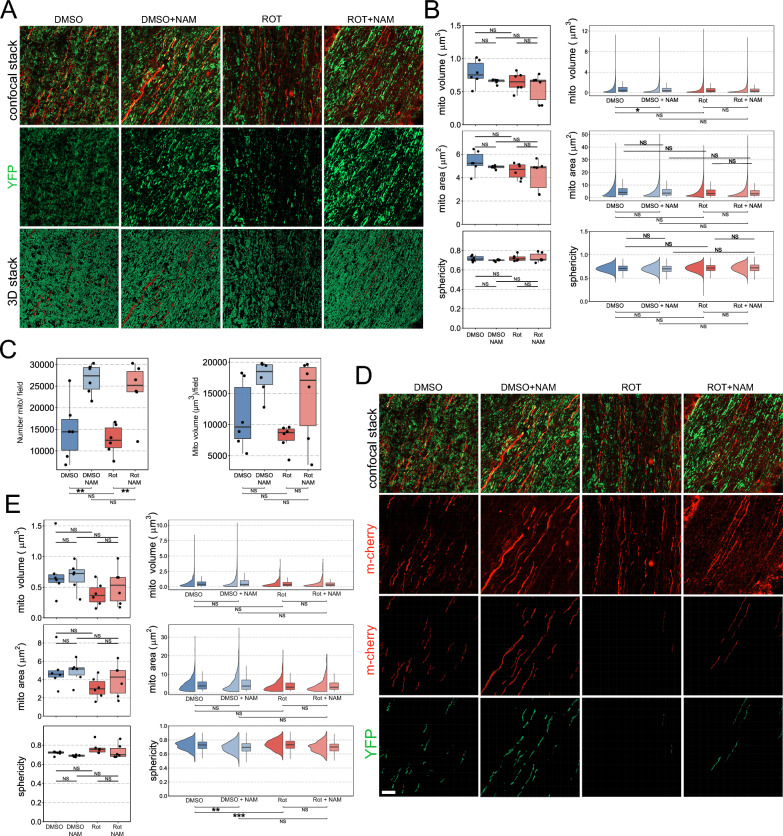
Nicotinamide increases total mitochondria within retinal ganglion cells axons in the optic nerve, which are not lost with complex I inhibition. (**A**) In MitoV mice, high resolution imaging of the optic nerve longitudinal sections allowed RGC specific YFP + mitochondria in RGC axons and somas to be captured for 3D reconstruction. (**B**) Morphological analysis revealed no significant changes in morphology induced by rotenone, with only a small increase in individual mitochondria volume in NAM treated, uninjured animals (DMSO + NAM). (**C**) Nicotinamide also significantly increased the density of RGC mitochondria in the optic nerve (number mitos/field) which remained high even with rotenone induced complex I inhibition. (**D**) To investigate mitochondrial changes at the level of individual RGC axons, we intravitreally injected an AAV2.2 encoding mCherry under a CMV promotor at low titer to sparsely label RGC axons. Individual mitochondria within these axons were reconstructed. (**E**) These mitochondria followed the same trend as for the optic nerve globally, but rotenone did induce a morphological change towards more spherical mitochondria, diverging from the typical prolate morphology of RGC axonal mitochondria. This did not occur in NAM treated animals, with uninjured NAM treated animals (DMSO + NAM) demonstrating that NAM promotes an exaggerated prolate morphology in the optic nerve. * *P* < 0.05, ** *P* < 0.01, *** *P* < 0.001, NS = *P* > 0.05

We next assessed retinal ganglion cell mitochondria in the optic nerve. GFP + mitochondria were reconstructed from longitudinal optic nerve sections, within the myelinated nerve, proximal to the eye (Fig. 2A). Global mitochondrial changes within in the optic nerve follow a different trend seen in the retinal ganglion cell components within the retina in which mitochondria did not demonstrate a significant morphological change following rotenone exposure (Fig. 2B). Nicotinamide treatment of control optic nerves resulted in a small but significant change in mitochondrial volume (Fig. 2B). We next analyzed optic nerve mitochondrial content *en masse*. In the control nicotinamide treated animals we observe a higher (~ twofold) total number of mitochondria per field of view (Fig. 2C). This again supports a hypothesis in which an increase in mitochondrial content provides neuroprotection. All together these results suggest that NAM pre-treatment provides resilience to rotenone-induced metabolic compromise by increasing the number of mitochondria, resulting in a threshold of mitochondria that buffer this stress.

In order to investigate mitochondrial changes at the level of individual retinal ganglion cell axons, we intravitreally injected an AAV2.2 encoding mCherry under a CMV promotor at low titer to sparsely label retinal ganglion cells. This results in strong, but sparse, expression of mCherry in axons along the optic nerve and therefore allows single axons or axon bundles to be followed along the optic nerve (Fig. 2D). mCherry positive retinal ganglion cell axon volumes within MitoV mice were used as a positive mask to isolate the mitochondria population present specifically within these individual axons from other mitochondria within the optic nerve (*i.e.* MitoV YFP within mCherry + axons) (Fig. 2D). The mitochondria within mCherry + axons follow the same trend as global GFP + mitochondria and do not demonstrate any significant volume and surface area change following rotenone exposure (Fig. 2E). However, mitochondria became significantly more spherical following rotenone exposure, and this was prevented by NAM pre-treatment (Fig. 2E). This suggests that across the optic nerve as a whole mitochondria shrink in response to rotenone, but the response is heterogenous at the level of individual axons, with some demonstrating earlier loss of elliptical morphology. These results suggest a greater effect of NAM on axonal mitochondria than on those in the retina.

### Nicotinamide prevents mitochondrial cristae loss at an ultrastructural level

The MitoV mice offer the benefit of a large *en masse* acquisition of a retinal ganglion cell-specific mitochondrial population, allowing the acquisition of thousands of mitochondria per field of view. Nevertheless, this *en masse* analysis does not allow a qualitative analysis of mitochondrial ultrastructure. In view of mitochondrial morphological changes detected in the MitoV mouse, we next aimed to assess if nicotinamide treatment can protect internal mitochondrial integrity. To determine this, we acquired transition electron microscopy images of mitochondria and quantified mitochondrial ultrastructure.

**Fig. 3:**
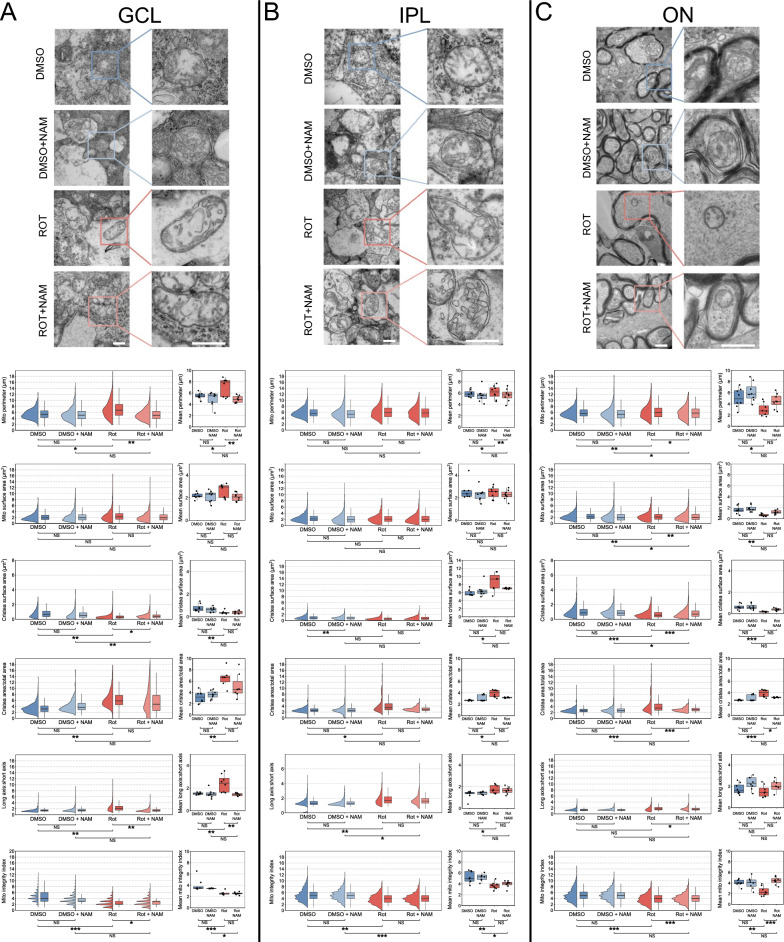
Nicotinamide prevents mitochondrial cristae loss at an ultrastructural level. TEM imaging of retinal cross sections allowed for reconstruction of mitochondria in (**A**) the GCL and (**B**) the IPL. TEM imaging of optic nerve cross sections allowed for reconstruction of mitochondria in (**C**) the optic nerve. (**A**) in the GCL rotenone induced an increase in mitochondrial perimeter, but with a loss of internal cristae structure, which was protected against by NAM pretreatment. (**B**) In the IPL, mitochondria demonstrated a subtle increase in size following rotenone injury, with an increase in cristae surface area. However, this occurred in addition to a loss of mitochondrial integrity index supporting a disorganization and fragmentation of internal cristae structure. NAM treatment did not significantly overcome this, suggesting that NAM may exert more benefit at the soma than in the dendrites. (**C**) In the optic nerve, mitochondria became significantly smaller with a loss of cristae structure and integrity. NAM was able to significantly protect against this. Scale bar = 100 nm

Mitochondria from all conditions were imaged across the different retinal ganglion cell components (soma, dendrites, axons) and ultrastructural morphology was assessed (Supplementary Fig. 1). In the GCL (somal mitochondria), rotenone exposure resulted in swollen and elongated mitochondria which was potently reversed by nicotinamide treatment (significant increase in mitochondrial outer perimeter, and the axis ratio relative to control; Fig. 3A). Subsequently, there was a decrease in the total cristae density, which was also prevented by nicotinamide treatment (significant increase of the total surface:cristae surface ratio, and decrease to the absolute cristae surface, and mitochondria integrity index compared to control) (Fig. 3A). In the IPL (dendritic mitochondria) rotenone treatment also resulted in more ovoid mitochondria with an increased cristae density, but in the absence of changes to overall size (significant increase in the axis ratio, relative to control, increase of the total surface:cristae surface ratio, and decrease to the mitochondria integrity index compared to control). This combination of increased cristae surface area and loss of mitochondrial integrity index supports a disorganization and fragmentation of internal cristae structure. Nicotinamide treatment did not prevent these changes other than limiting the increase in cristae surface area relative to mitochondrial area (Fig. 3B), suggesting that NAM may exert more protective effects at the soma than in dendrites. In the optic nerve (axonal mitochondria), following rotenone exposure, mitochondria become significantly smaller with a lower cristae density (significant decrease in perimeter, surface, total surface:cristae surface ratio, and mitochondria integrity index compared to controls) which was potently protected by nicotinamide treatment (Fig. 3C). Collectively, these data suggest that effects of Complex I inhibition are not uniform across retinal ganglion cell compartments, and that nicotinamide prevents these changes independently.

## Discussion

Metabolic decline may be a critical, and treatable, pathogenic component of neurodegenerative disease. Given the high metabolic requirements of the retinal ganglion cell, its unique anatomy, and its constant stressors (*e.g.,* intraocular pressure, blue light, changes in vascular tone), the retinal ganglion cell is a uniquely challenged neuron that is highly susceptible to bioenergetic failure [4]. A role for mitochondrial dysfunction has been well-demonstrated in mitochondrial optic neuropathies (*e.g.,* LHON, ADOA [18, 26]) and other primary blinding diseases (*e.g.,* glaucoma [27]). Although these diseases are well identified, the treatment of these monogenic mitochondrial diseases consists mainly of genetic counselling and multi-disciplinary medical and para-medical teamwork aimed at minimizing the morbidity. There are no long-term protective therapies and, as such, neuroprotective strategies are of great therapeutic need.

We have previously discovered metabolic dysfunction and mitochondrial abnormalities occurring prior to neurodegeneration in glaucoma (in glaucoma patients and glaucoma animal models [5, 10, 13]). A major finding from these studies was an age- and/or intraocular pressure- dependent decline in NAD [5, 10], an essential metabolite for neuronal health. Retinal ganglion cells primarily generate NAD through the NAD salvage pathway which initiates with nicotinamide before undergoing a two-step process to become NAD [28]. Preventing this NAD depletion by increasing the NAD salvage pathway precursor nicotinamide provides a robust neuroprotection in glaucoma animal models [5, 10] and can improve visual function in existing glaucoma patients [16, 17]. Long-term RCTs for nicotinamide in glaucoma are now ongoing in Sweden, Australia, Singapore, and the UK. Given the potential for nicotinamide in treating metabolic dysfunction in glaucoma, it could be a valid approach in treating other mitochondrial optic neuropathies. To assess this in LHON, a maternally inherited blinding disease that predominantly affects young men, we used a Complex I inhibition model in which intravitreal rotenone injection results in the rapid loss of retinal ganglion cells in the inner retina. Pre-treatment with 500 mg/kg/d (~ 2 g/d in human, and two-thirds of the glaucoma clinical trial dose) prior to rotenone exposure resulted in modest retinal ganglion cell survival and a partial reversal of the mitochondria phenotype.

In this study we performed two complementary techniques to assess retinal ganglion cell mitochondria. To assess mitochondria *en masse* we leveraged a new mitochondria report mouse, MitoV [10]. Neurons in these mice express YFP driven by a rat neuron-specific *Eno2* promoter. Mitochondrial localization is obtained by fusing the cytochrome *c* oxidase subunit 8A (Cox8a) sequence to the YFP N-terminal resulting in YFP tagged mitochondria [29]. From a number of founder mice, we identified a founder in which YFP was specific to retinal ganglion cells in the inner retina. A major benefit in these mice is the capacity to quantitatively study the global mitochondrial distribution in retinal ganglion cells during pathological processes and to simultaneously acquire a large number (100,000 s) of mitochondria per condition and treat these images in a semi-automated manner using a 3D reconstruction software. The MitoV mouse also presents an advantage in that expression is retinal ganglion cell specific in the inner retina and YFP can be tracked down the optic nerve, and that the YFP is not reliant on mitochondrial function (and, as such, all mitochondria are tagged regardless of their metabolic state). To assess mitochondrial ultrastructure at the individual mitochondria level we used TEM, and the findings here broadly support the findings from the MitoV mouse with the extension to mitochondrial cristae analysis. In both cases, nicotinamide provides a compartmentally dependent neuroprotection by limiting mitochondrial gross structural changes and internal cristae disruption. Rotenone exposure resulted in differing mitochondrial responses within the soma, dendrites, and axons of retinal ganglion cells. In glaucoma, this compartmentalized degeneration of retinal ganglion cells has been well established [30–34]. In the GCL, we observed that rotenone induced a swelling of mitochondria *en masse* (in MitoV) which was confirmed at an ultrastructural level (TEM) and occurred in combination with a loss of cristae content. In the IPL, there was a significant loss of mitochondria, and in those surviving, mitochondrial size did not significantly change but cristae content was lost. In the optic nerve, loss of mitochondria was most pronounced, and those surviving were typically significantly smaller and less internally complex. These differences may reflect the differing dynamics of mitochondria within the soma, dendrites, and axons (*e.g.,* in turnover) and the differing metabolic pressures they may face [4, 35, 36]. In the GCL and optic nerve, nicotinamide treatment was robustly protective against mitochondrial changes whereas in the IPL the protection afforded was weaker. This may be related to the varying NAD-dependent mechanism within these separate compartments (*e.g.,* axon degeneration is tightly coupled to mitochondrial integrity and local NAD levels [37]). Further understanding of how NAD partitions and is maintained within these compartments, especially when its NAD capacity is increased through nicotinamide supplementation, will be important to understand retinal ganglion cell degeneration and neuroprotection.

*Limitations* Although the MitoV transgenic mouse presents many benefits in quantitative analysis, we encountered a technical limitation using the fluorescent microscopy. The irregular YFP stability under long confocal microscopy acquisition does not allow imaging of the YFP native signal without bleaching when acquiring z-stack volumes. To address this, we used an anti-GFP antibody, which normalizes the YFP signal, stabilizes, and allows for longer scanning sessions and thus increases the potential resolution of the image. As a complementary technique to the super resolution fluorescent microscopy, we used TEM which offers a substantially higher resolution and none of the noise / bleaching bias introduced by antibody labelling. Unfortunately, to assess many mitochondria in this way, it is only feasible to perform 2D analysis, and not 3D EM (*e.g.,* SBFSEM which allows for 3D reconstructions but at the cost of throughput). Rotenone is often used to model neurodegeneration, and in our hands, results in a loss of 50–60% of retinal ganglion cells within the first 24 h. Although highly reproducible, the absence of a longer timeframe limits its utility to model the human disease in which, once initiated, vision loss takes place over several months. The results at least strongly demonstrate that nicotinamide can limit the degeneration induced by a severe-acute mitochondrial stress and support its testing in other animal systems. Given nicotinamide’s long history and robust safe profile, testing in LHON patients could be encouraged within a defined and controlled clinical setting.

### Supplementary Information

**Additional file1**: **Fig. 1** Representation of the long:short axis measurement (*yellow:blue*), cristae surface area (*red*), and mitochondrial perimeter (green).

## Data Availability

All data generated is presented within the manuscript. Raw datasets are available from the corresponding author on reasonable request.

## Abbreviations

CMT: Charcot-Marie-Tooth
DMSO: Dimethyl sulfoxide
GCL: Ganglion cell layer
IOP: Intraocular pressure
IPL: Inner plexiform layer
NAM: Nicotinamide
ON: Optic nerve
ONH: Optic nerve head
RBPMS: RNA-binding protein with multiple splicing
RGC: Retinal ganglion cells
ROT: Rotenone
YFP: Yellow fluorescent protein
