# Supplementary material for: Prophylactic nicotinamide treatment protects from rotenone-induced neurodegeneration by increasing mitochondrial content and volume
Source: Acta Neuropathol Commun. 2024 Mar 1;12:37. doi: 10.1186/s40478-024-01724-z (PMC10908050; doi:10.1186/s40478-024-01724-z)
Supplement: Supplementary file 1 — Additional file1: Fig. 1 Representation of the long:short axis measurement (yellow:blue), cristae surface area (red), and mitochondrial perimeter (green). [file 40478_2024_1724_MOESM1_ESM.docx]

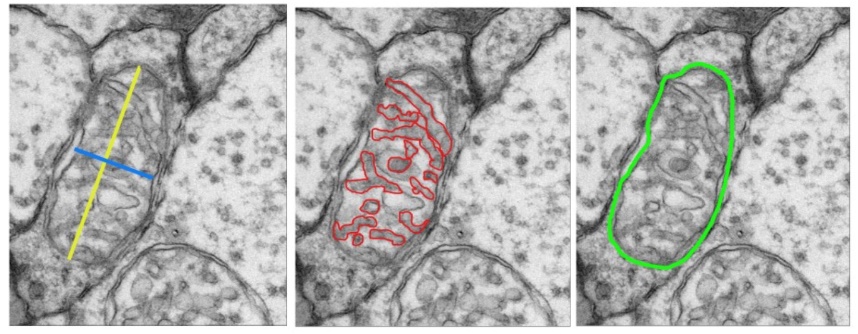


**Supplementary Figure 1.** Representation of the long:short axis measurement (*yellow:blue*), cristae surface area (*red*), and mitochondrial perimeter (green).
